# Artificial Neural Network Approach for Modeling of Effect of Ultrasound on the Dissolution of Magnesia in Aqueous Carbon Dioxide

**DOI:** 10.1021/acsomega.3c03668

**Published:** 2023-11-16

**Authors:** Fatih Sevim, Ahmet Atalay

**Affiliations:** † Engineering Faculty, Department of Chemical Engineering, Atatürk University, Erzurum 25030, Turkey; ‡ Engineering Faculty, Department of Civil Engineering, Atatürk University, Erzurum 25030, Turkey

## Abstract

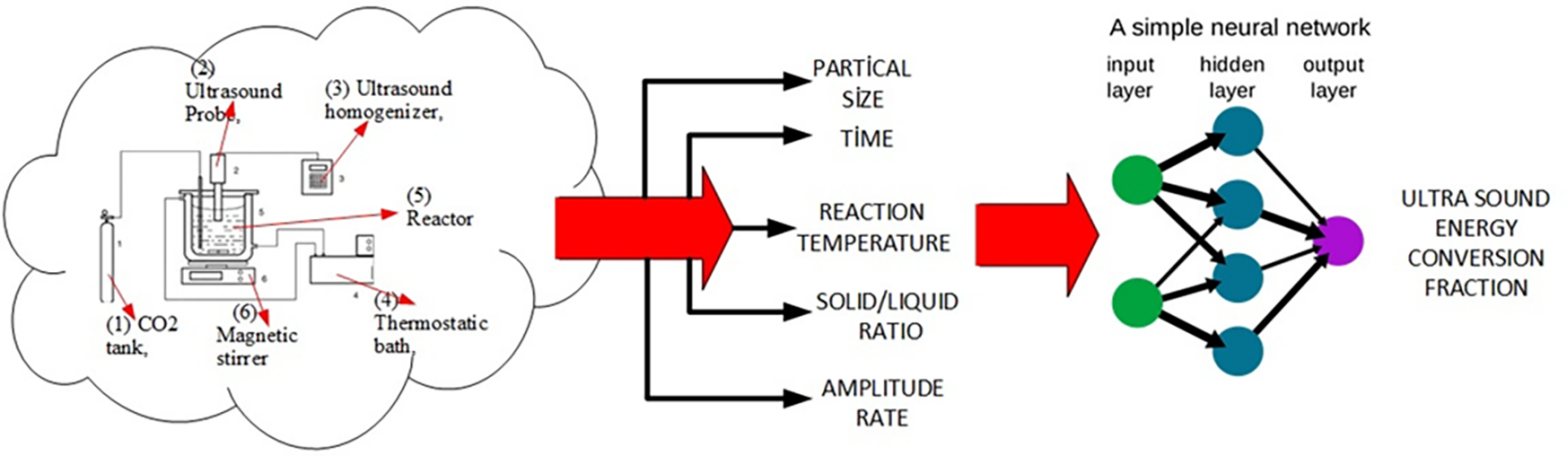

This article is about dissolving magnesia in aqueous carbon dioxide by applying ultra sound. Particle size, reaction temperature, and solid/liquid ratio were chosen as the experimental parameters. As a result of the experimental study, the ultrasound energy conversion fraction (USECF) was obtained. Using experimental data, a model has been created for artificial neural networks and USECF. Created and modeled, the particle size, time, reaction temperature, solid/liquid ratio, and amplitude rate were determined as input variables. USECF was determined as the output variable of the model. In this study, six different ANN models were created by using two different learning algorithms and three different transfer functions. The results of these models were compared with the experimental results. It has been determined that the model established with the Levenberg Marquart learning algorithm and the TANSIG transfer function gives the best result of the ANN model compared to the other models. The ANN model established with the Gradient Descent learning algorithm and the LOGSIG transfer function were determined to be the second model that gave the best results. The regression *R* value for the model performance indicator training data was determined as 0.99 after validation, and the regression *R* value for the test data was determined as 0.99.

## 1 Introduction

Magnesium compounds are used extensively for refractories and insulating compounds as well as in the manufacture of rubber, printing inks, pharmaceuticals, and toilet goods. Magnesite ore is the primary source for the production of magnesium compounds. The natural magnesite contains impurities such as silicon, iron, and calcium, which affect the quality of products. Therefore, these impurities must be removed from the ore. For this purpose, some physical and chemical beneficiation methods are used.^1^ For the selective leaching of magnesium, the ore must be calcined to give caustic calcined magnesia (calcined magnesite) before leaching. Since the caustic calcined magnesia has some favorable physical properties such as high reactivity and porosity, it can easily be reached by mild and weak reactants. Therefore, the calcinations and its conditions are important for the purification of magnesite by a calcinations leaching proces.^2^

Ultrasound is known to have great effects on chemical reactions.^3^ Unlike other new technologies, which require some special attributes of the system being activated in order to produce an effect, e.g., the use of microwaves (dipolar species), electrochemistry (conducting medium), and photochemistry (the presence of a chromophore), ultrasound requires only the presence of a liquid to transmit its energy.

Ultrasound causes high-energy chemistry. It does so through the process of acoustic cavitation: the formation, growth, and implosive collapse of bubbles in a liquid. During cavitation collapse, intense heating of the bubbles occurs. These localized hot spots have temperatures of roughly 5000 °C, pressures of about 500 atm, and lifetimes of a few microseconds. Shock waves from cavitation in liquid–solid slurries produce high-velocity interparticle collisions, the impact of which is sufficient to melt most metals. Applications to chemical reactions exist in both homogeneous liquids and liquid–solid systems. Of special synthetic use is the ability of ultrasound to create clean, highly reactive surfaces on metals. Ultrasound has also found important uses for the initiation or enhancement of catalytic reactions, in both homogeneous and heterogeneous cases.^4^

In studies carried out in recent years, the dissolution of ores has been accelerated by using different techniques. One of the techniques used for this purpose is ultrasound energy. It has been known for many years that ultrasound energy has a great application area in many chemical and industrial processes. Application areas of ultrasound energy are as follows: cleaning, sterilization, flotation, drying, gasification, defoaming, soldering, plastic welding, drilling, filtration, homogenization, emulsification, dissolution, biological cell division, extraction, crystallization, and chemical reaction stimulants. While many studies were carried out on biological effects in the 1960s,^5^ studies began to be conducted on physical and chemical effects after 1990.^6^ Especially in recent years, studies examining the effect of ultrasound energy on organic, inorganic, and organometallic sonochemistry have been conducted,.^7,8^ With the development of artificial intelligence techniques, artificial neural network models have been developed to predict the results obtained from experimental studies. Sun et al.^9^ used neural networks and ultrasound for quantifying the dispersion of mineral filler in a polymer. In the study, it was attempted to build a neural network model to predict the filler dispersion index. Rajković et al.^10^ compared the use of ANN with the topology 4–10–1 with the response surface methodology (RSM) for ultrasound-assisted sunflower oil transesterification using a KOH catalyst. Badday et al.,^11^ performed transesterification of crude Jatropha oil to fatty acid methyl esters in an ultrasound assisted process in the presence of different heteropolyacid-based catalysts. The experimental data were then used to construct an artificial neural network (ANN) model to predict the response of the reaction. Banerjee et al.^12^ used ANN models to predict the percentage removal of Cr(VI).

The aim of this study is to examine the effect of ultrasound energy on the dissolution of calcined magnesite ore with CO_2_ in an aqueous medium. It is also to create a prediction model using artificial neural networks (ANN) for the ultrasound energy conversion fraction. In this study, six different ANN models were created using two different learning algorithms and three different transfer functions.

## 2 Experimental Section

The material of this study is the data obtained from the experimental study. The leaching temperature, leaching time, particle size, solid/liquid ratio, amplitude, velocity, and ultra sound energy conversion fraction values used in the experimental study were determined.

### 2.1 Experimental Study

The magnesite ore used in the study was provided from the region of Oltu, Erzurum, Turkey. The ore was crushed and sieved by ASTM standard sieves to give −25 + 35, −35 + 50, −50 + 70, and −80 + 100 mesh size fractions for calcination experiments. Calcination experiments were made at 700 °C. The composition of the original magnesite ore used in the experiments is given in Table 1.

**Table 1:** Chemical Composition of Magnesite Ore

component	by weight. %
MgO	45.95
CaO	1.40
Fe_2_O_3_	0.75
SiO_2_	1.98
loss on ignition	50.15

**Figure 1:**
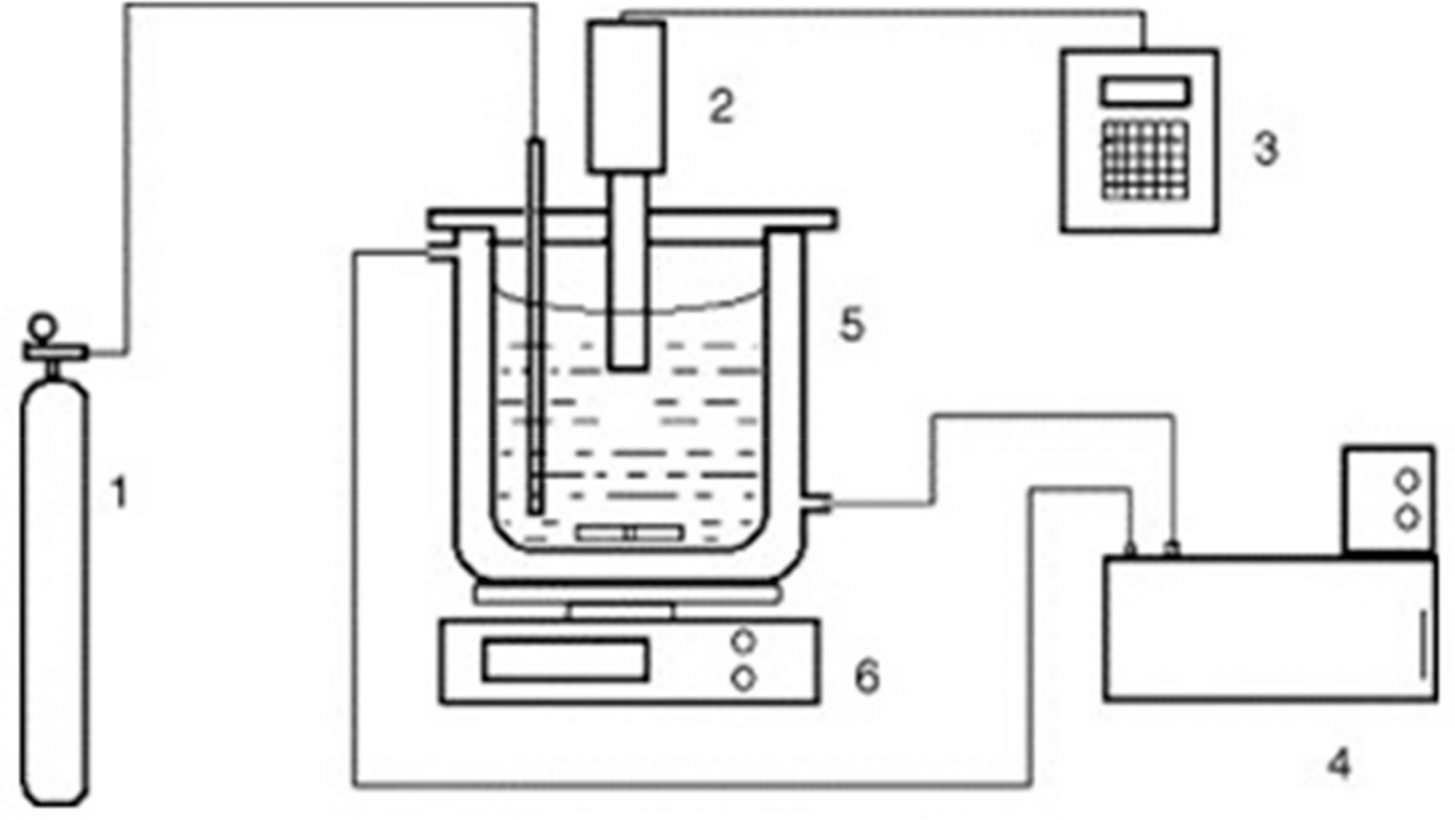
Scheme experimental setup for dissolution. (1) CO_2_ tank, (2) ultrasound probe, (3) ultrasound homogenizer, (4) thermostatic bath, (5) reactor, and (6) magnetic stirrer.^13^

The setup for dissolution experiments is shown in Figure 1. The experimental setup was carried out in a jacketed glass reactor with a volume of 500 mL, as given in Figure 1. It consists of an ultrasonic generator (Cole Parmer, Ultrasonic homogenizer, 400 W, 20 kHz), a probe with a tip radius of 1 cm, and a thermocouple. The probe was covered with a Teflon band for hindering the probable corrosion of the probe in H_2_CO_3_.

The leaching studies were carried out in a jacketed glass reactor. The reaction temperature was controlled with a thermostatic bath. The leaching experiments were conducted under atmospheric pressure conditions. The CO_2_ gas (97%) was supplied to the reactor from a CO_2_ cylinder, and the gas was bubbled from the bottom of the reactor by means of a disk type gas disperser. First, a heating reactor containing 250 mL of distilled water to the desired reaction temperature carried out the leaching experiments. After feeding CO_2_ to the reactor for 15 min to obtain a saturated solution, a 2.0 g sample of magnesia was added and stirred mechanically. CO_2_ was continuously fed into the reactor during leaching to maintain saturation. Thus, the pH of the slurry was kept constant. Solution samples were taken at intervals of time and filtered immediately, and the Mg^2+^ content was analyzed by a volumetric method.^14^

#### 2.1.2 Features of Ultrasound Device

For the dissolution of calcined magnesite with CO_2_ in an aqueous medium, at 400 W and 20 kHz, a Cole Palmer Ultrasonic Homogenizer brand ultrasound device with a 1 cm diameter probe was used.

There are two generally used methods to measure the amount of ultrasonic power reacting in ultrasound devices. The most widely used system is the calorimetric method, which measures the initial heat rate produced when the system is emitted by ultrasound power. The other method is systems with chemical dosimeters that monitor the sonochemical production of the chemical species. In this study, the power of the ultrasound device was determined using the calorimetric method and the Weissler reaction.^15^ From the obtained results, it is stated that the Weissler reaction is directly and linearly related to the calorimetrically determined ultrasonic power. For this process, after the reactor was completely isolated, it was filled with 400 mL of distilled water at a constant temperature, and the final temperature of the water was determined by applying ultrasound energy at a certain amplitude for 15 min. Then, the process was repeated by changing the amplitude rate of the ultrasound energy. The power amount of the ultrasound energy was calculated, and the results are given in Table 2.^13^

**Table 2:** Power Amounts of Ultrasound Device at Different Amplitude Rates

amplitude rate (%)	power amount (Watt)
20	10.96
30	16.43
40	21.91
50	27.39

**Table 3:** Parameter Values Used in Experiments

parameter	selected values
leaching temperature (°C)	14; 20; 30; 40
leaching time (dk)	3; 5; 10; 15; 30; 45; 60; 75
particle size (mesh)	–25 + 35; −35 + 50; −50 + 70; −80 + 100
solid/liquid ratio (g/g)	1/250; 2/250; 4/250; 8/250
amplitude rate (%)	20; 30; 40; 50

**Figure 2:**
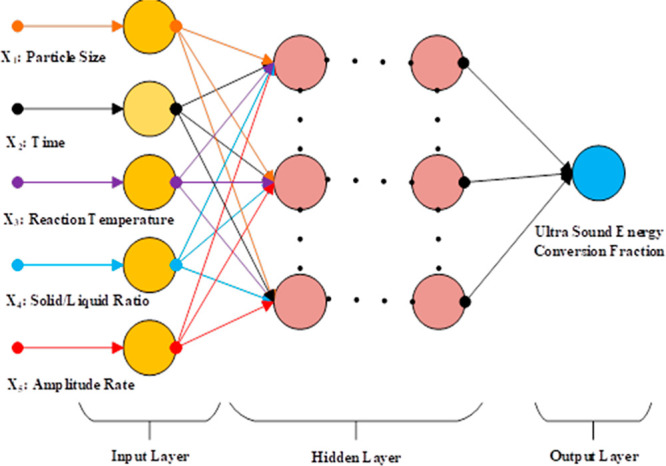
ANN model.

**Figure 3:**
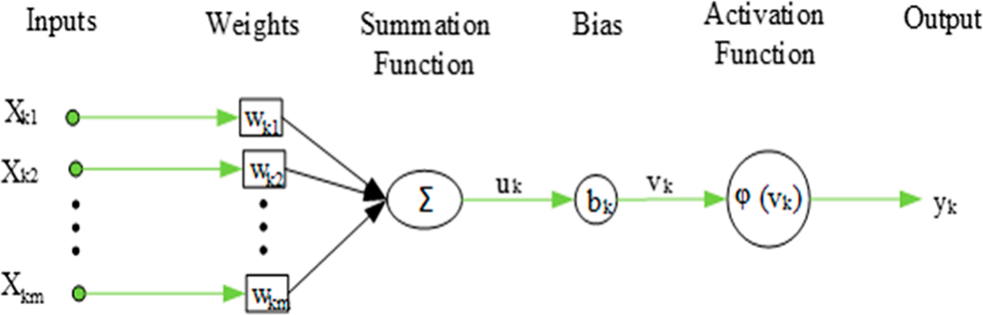
Structure of neuron cell.

**Table 4:** Data Set Descriptive Statistics

variable name	*N*	range	minimum	maximum	mean	std. deviation
particle size	110	15.00000	10.00000	25.00000	19.1818182	3.14243468
time	110	72.00000	3.00000	75.00000	24.4363636	22.05270055
reaksion temperature	110	26.00000	14.00000	40.00000	21.2000000	5.35835111
solid/liquid ratio	110	0.02800	0.00400	0.03200	0.0101455	0.00656210
amplitude rate	110	30.00000	20.00000	50.00000	45.7272727	9.13297191
ultra sound energy conversion fraction	110	0.93616	0.06367	0.99983	0.5176232	0.31991790

**Table 5:** Properties of ANN Models

				R			
model	transfer function	epoch	mean square error	training data	validation data	test data	all data
Levenberg–Marquardt- LM1	TANSIG	200	0.00068069	0.998	0.998	0.995	0.998
Levenberg–Marquardt-LM2	PURELIN	200	0.018657	0.940	0.960	0.920	0.940
Levenberg–Marquardt-LM3	LOGSIG	500	0.00045522	0.996	0.995	0.998	0.996
gradient descent-GD1	TANSIG	350	0.00013153	0.964	0.998	0.999	0.972
gradient descent-GD2	PURELIN	150	0.0050043	0.944	0.932	0.946	0.942
gradient descent-GD3	LOGSIG	200	0.0015447	0.996	0.993	0.996	0.995

**Figure 4:**
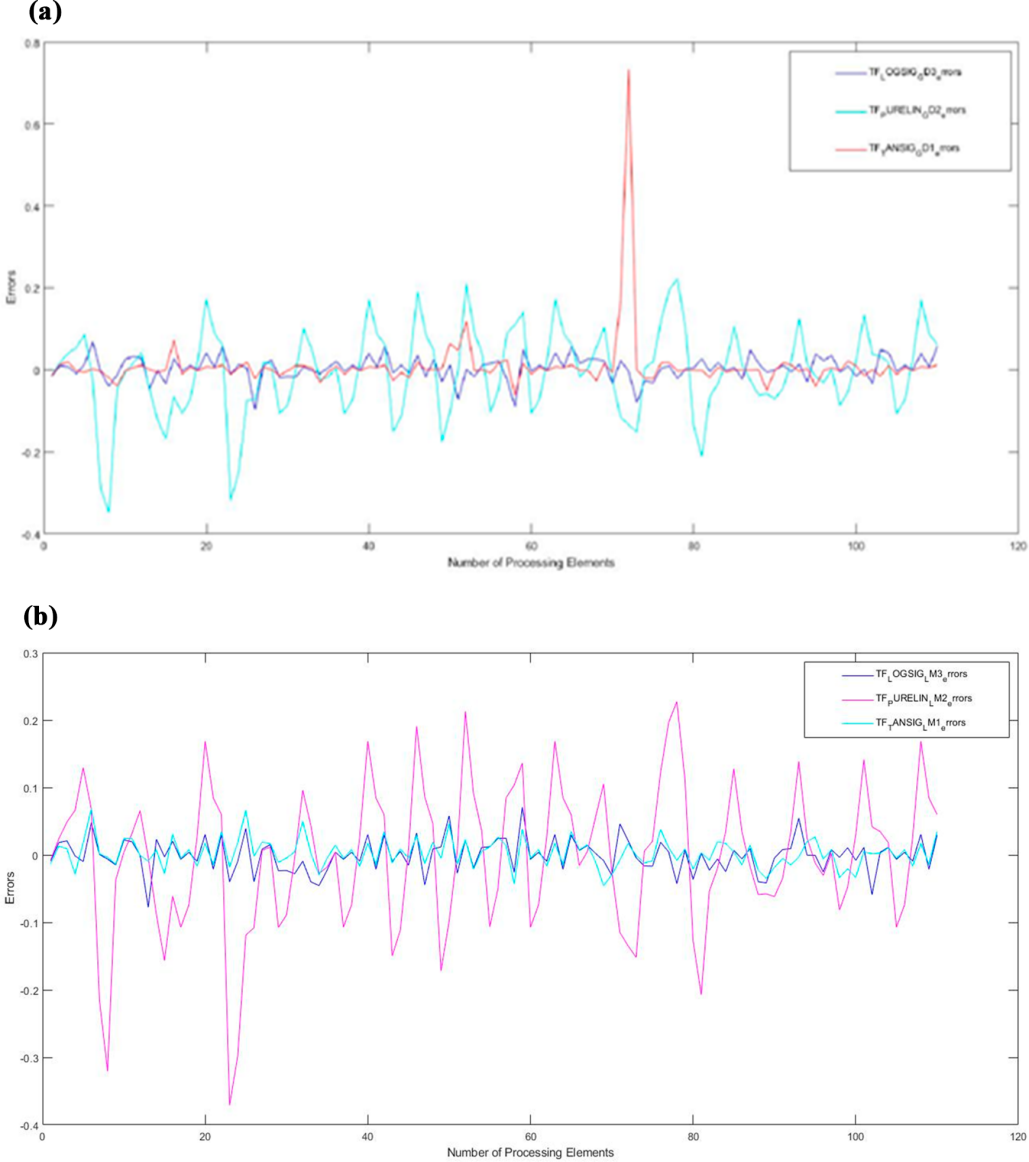
(a) Gradient descent learning algorithm error values. (b) Levenberg–Marquardt learning algorithm error values.

Faid et al.^16^ compared the effect of ultrasound in different reactors at 20 kHz. For these horn type pipes, a quarter of horn type pipes and a tube were used, and the effects of cavitation in them were investigated. The crosstalk and radial profiles of the mass transfer coefficients were investigated in three devices at various power signals. With or without a constant fluctuation, large differences were detected in all of these experiments. In another study, standard methods such as chemical dosimetry, use of a thermal receiver, and use of a electrochemical probe were compared for local measurements of ultrasound power effects in a reactor.^17^ It has been observed that ultrasound changes the conditions outward from the tube axis. The results were also replicated with thermal and electrochemical probes.

A mechanical stirrer with adjustable speed was used for mixing a constant temperature circulator to keep the reaction temperature constant, and an ultrasound device was used to examine the effect of ultrasound. It was observed that the CO_2_ gas fed to the reactor dissolves the magnesite ore. In the studies, magnesite dissolution was observed under reaction temperature, particle size, solid/liquid ratio, ultrasound amplitude, rate, and time parameters. These selected parameter values are given in Table 3.

### 2.2 Artificial Neural Network Modeling

Artificial neural networks (ANNs) are powerful mathematical modeling tools designed for complex systems. Since the 1940s, ANN methods have been successfully applied in different areas of engineering and science.^18,11^

ANN can provide a way for modeling the relationship between measured and controlled parameters of a complex process without the need of a thorough understanding of the process itself.^19^ On the other hand, ANNs are data processing models produced with various mathematical and electrical methods based on the physiological structure of the brain. Due to the parallelism, learning, and adaptive features of ANN, it has found widespread application as a data processing system. ANN is a data processing system that is not algorithmic and numerical but capable of parallel processing. In brief, ANNs are interconnected artificial neural cells.^20^

The neural network is a parallel distributed processor consisting of simple processing units called neurons, which have tendencies for storing experimental knowledge and making it ready to use. ANN resembles the human brain in two respects, i.e., gaining knowledge from its environment by learning processes and storing the acquired knowledge using the strength of interneuron connections known as synaptic weight. The process used to conduct the learning process is called the learning algorithm, which has the function of modifying the weights via a systematic fashion to address the required purposes.^21^

In ANN modeling, the data collected from the experimental yield values for the three catalysts were used for network training to establish the network model that could compute the predicted yield values from the input reaction conditions using MATLAB R2018 b software. All experimental data were divided randomly into three groups, i.e., training (70%), validation (15%), and testing data (15%).

The number of neurons was optimized between 3 and 20 neurons in the hidden layer by applying the training algorithms in this study. The learning algorithms that are frequently used in studies in the literature are Levenberg–Marquardt and gradient descent.^22−26^

A Levenberg–Marquardt back-propagation algorithm was designed to approach the second-order training speed without the need for calculating the Hessian matrix as the matrix is approximated to the simplest form. The Jacobian matrix, which consisted of the first derivative of the network error was computed by a back-propagation method used in the approximation as it was much less complex than the Hessian matrix.^27^ The gradient descent strategy used in the back-propagation technique has the advantage of a fast convergence speed. It is suitable for problems with large scale, but it converges closer to the initial point rather than converging to the global optima. In this study, Levenberg–Marquardt and Gradient Descent learning algorithms were used to create ANN models.^28^

In this study, the proposed ANN model architecture for Ultrasound Energy Conversion Fraction is presented in Figure 2. Particle size, time, reaction temperature, solid/liquid ratio and amplitude ratio were used as input parameters of the models, and Ultra Sound Energy Conversion Fraction was used as output parameter.

The general structure of the model consists of the input layer, the hidden layer, the output layer, and the output. The model has five inputs and one output. The hidden layer of the model is composed of ten layers, and the output layer is composed of one layer.

In an artificial neural network, the basic processor is a neuron. A neuron consists of five parts: input, weights, summation function, bias, activation function, and output.^29,30^ In general, the structure of the artificial nerve cell consists of five parts (Figure 3).

## 3 Results and Discussion

ANN modeling of multilayer perceptron with Gradient Descent and Levenberg–Marquardt algorithms has been tried using three different standard transfer functions in a single hidden and output layer. Figure 2 presents the schematic flow diagram of the ANN used. The input variables are the particle size, time, reaction temperature, solid/liquid ratio, and amplitude rate, and the output variable is the Ultra Sound Energy Conversion Fraction. Bar and Das^22−25^ and Bar et al.^26^ have demonstrated successful predictability using ANN even without normalization. Hence, in the present cases, the original data have been used for ANN modeling. In this study, input and target variables and their descriptive statistics are shown in Table 4. Out of the data points, 70, 15 and 15% data are used for training, cross-validation, and final prediction.

In this study, two different learning algorithms as Gradient Descent and Levenberg–Marquardt were used. Three different transfer functions in the Matlab neural network toolbox were used for each learning algorithm. The statistics about the networks created in the study, the properties of the parameters of the networks, and the data sets used are given in Table 5 below.

The good performance of the network is dependent on the value of statistical parameters like MSE and cross-correlation coefficient (*R*). These values should be as small as possible. The cross-correlation coefficient value should be close to unity for better predictability.

Figure 4a and b show the variation of minimum value of error with the number of nodes for different transfer functions. In models created with three different transfer functions in two different learning algorithms, the best performance is the Gradient Descent third model and the first model with Levenberg–Marquardt.

**Figure 5:**
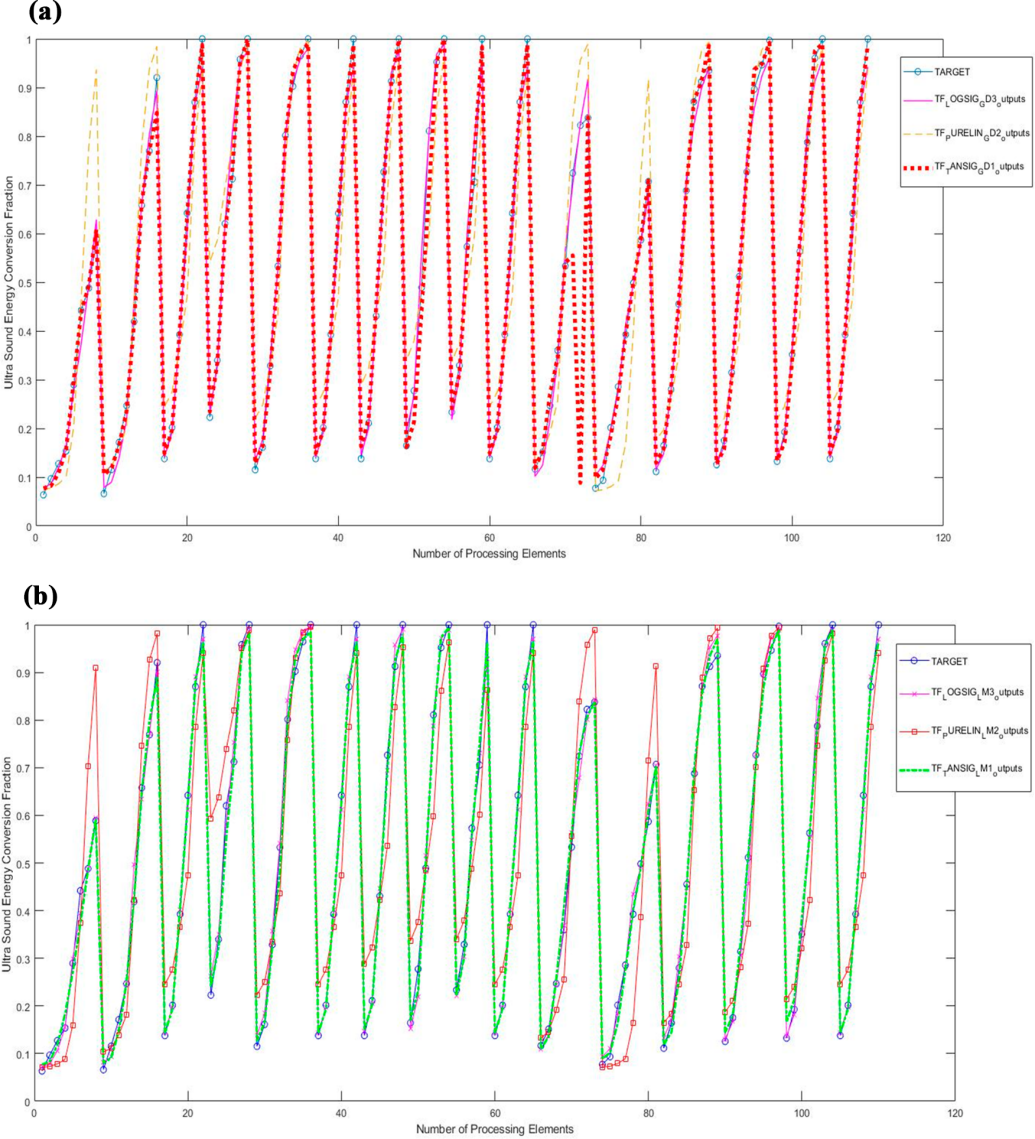
(a) Results of gradient descent algorithm models. (b) Results of Levenberg–Marquardt algorithm models.

A comparison of the results of the ANN models established in this study with the actual result values was obtained as shown in Figure 5a and b.

A visual comparison of the results and target values of the models created by using two different learning algorithms and using three different transfer functions is shown in Figure 5a and b.

When the model results and the changes in the target values were compared, it was determined that the LM1 and GD3 models were the best models.

In ANN models, the independent variables are called inputs, and dependent variables are called outputs. Operations were done using the Matlab program. Different architectures, training algorithms, transfer functions, and initial weighting coefficients were tested during the training of the ANN. Thus, we tried to determine the network architecture, training algorithm, and transfer function that gave the best results.

Data sets were divided into three training, testing, and validation sets. Seventy percent of the data set was used to train the network, while the remaining 30% was employed for testing and validation. The validation set was used to train the network. The test data set is not used in training at all, and it is designed to give an independent assessment of the network’s performance when the entire network design procedure is completed. If the performance of the trained data set is very low, whereas the performance of the validation data set is large, then memorization of the network is suspected. In such a case, the network must be retrained.^31,32^ Training algorithms do not use validation or test sets to adjust network weights. The performance of the training, validation, and test sets is the standard deviation ratio of that set. In other words, it is the ratio of the standard deviation of the errors to the standard deviation of the actual values and is an important performance criterion in the training phase.^32^

As a performance criterion, the regression *R* value and the mean of the error squares were considered together with the MSE. The closer the mean of the square of the errors between the target data and the output values of the model, the higher the performance of that model. Similarly, the regression value between the target data and the output values of the model is close to 1, indicating that the performance of the model is good. According to the results of the ANN model, the Levenberg–Marquardt backpropagation algorithm was the best training procedure that achieved the highest *R* value.

**Figure 6:**
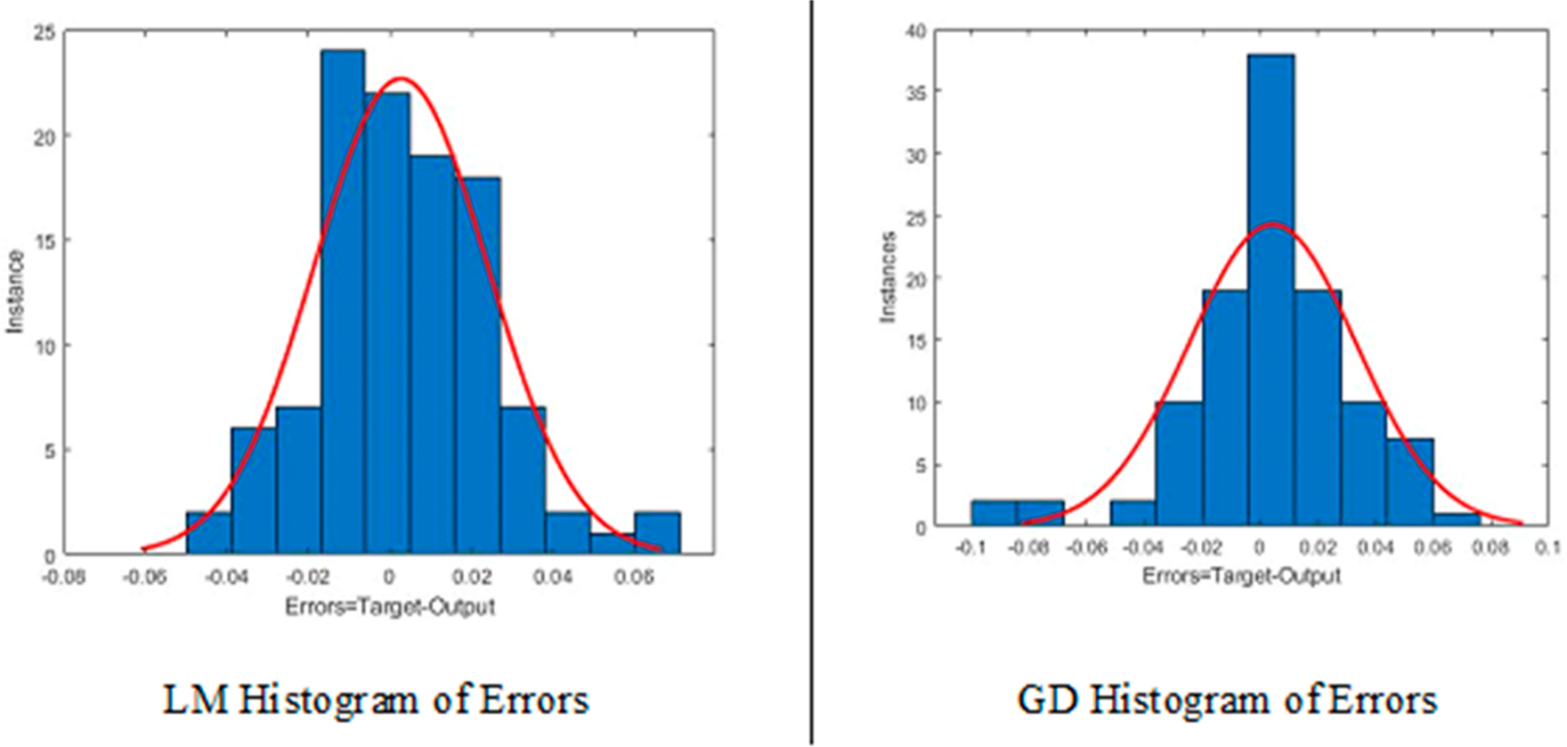
Histogram of errors.

While both the Leveneberg-Marquart algorithm and Gradient Descent algorithm were applied in the training of the network, the best result was achieved with 20 000 iterations. A histogram graph of the errors of the network created in the study was obtained as that in Figure 6.

When Figure 6 is examined, it is seen that the errors are normally distributed, the distribution of the estimated values and errors is close to the 0 (zero) line, the error histograms are open to the right and left, but the zero error frequency is high, and there is a good correlation between the predicted values and the observed values. It appears that there is compatibility.

The general equation of the model is specified in eq 1.
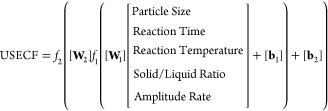
1

The values of **W**_1_, **W**_2_, **b**_1_, and **b**_2_ matrices are taken after the learning process of the network is completed. To estimate the USECF, each variable needs to be normalized using product coefficients and constants. Eq 1 represents the activation functions as *f*_1_ and *f*_2_, which are used for normalization. Generally, the most commonly used activation functions are linear (linear), sigmoid, bipolar sigmoid, and hyperbolic tangent activation functions.^33^ In this study, *f*_1_ is the hyperbolic tangent activation function, and *f*_2_ is the sigmoid activation function. These functions are specified in eqs 2 and 3.
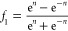
2
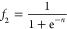
3

In eqs 2 and 3, *n* represents the net number of entries in the network. The results of the ANN model are calculated according to the equation in eq 1.

**Figure 7:**
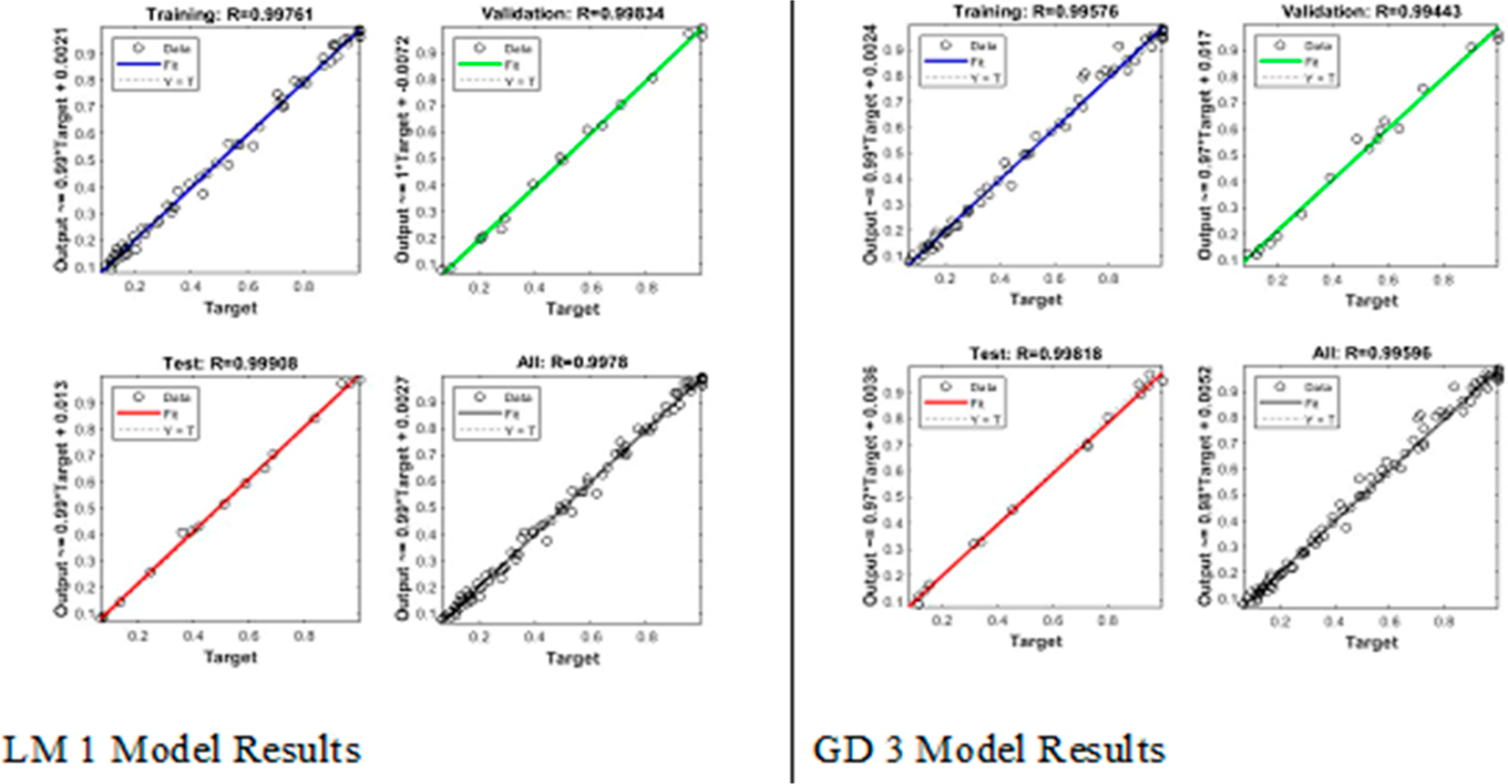
Model results.

Regression graphs showing the relationship between the data sets used in the study and the outputs of the model obtained are shown in Figure 7.

In the study, regression *R* values were obtained for the training, validation, and test data sets, showing the relationship between the model and the results. Correlation coefficients for training, validation, and test data were determined as 0.99, 0.99, and 0.99, respectively. When all data and model results were compared, the regression *R* value was determined as 0.99 (Figure 7).

The descriptive statistics of the data and ANN model results used in this study are listed in Table 6. According to the mean standard error values, it is understood that the ANN model results are very close to the experimental results (Table 6).

**Table 6:** Data Set Statistics

descriptive statistics								
Varible Name	N	range	minimum	maximum	mean		std. deviation	variance
	statistic	statistic	statistic	statistic	statistic	std. error	statistic	statistic
Particle_Size	110	15.00000	10.00000	25.00000	19.1818182	0.29961939	3.14243468	9.875
Time	110	72.00000	3.00000	75.00000	24.4363636	2.10264250	22.05270055	486.322
Reaction_Temperature	110	26.00000	14.00000	40.00000	21.2000000	0.51089873	5.35835111	28.712
Solid_Liquid	110	0.02800	0.00400	0.03200	0.0101455	0.00062567	0.00656210	0.000
Speed	110	30.00000	20.00000	50.00000	45.7272727	0.87079470	9.13297191	83.411
Target	110	0.93616	0.06367	0.99983	0.5176232	0.03050297	0.31991790	0.102
LM1 Output	110	0.91766	0.07652	0.99418	0.5149702	0.03025231	0.31728893	0.101
GD3 Output	110	0.91018	0.07791	0.98809	0.5132933	0.03006152	0.31528784	0.099

**Figure 8:**
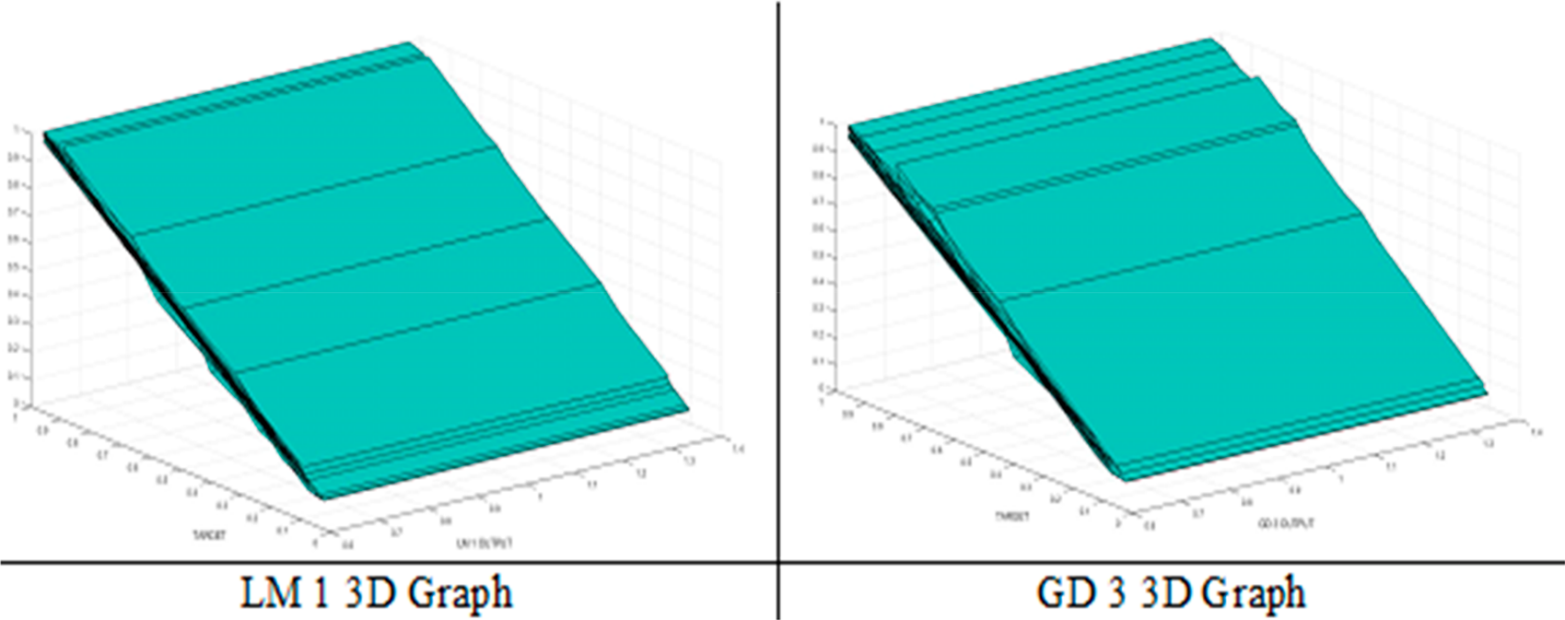
3D graph of the best ANN model results.

A comparison of ANN model results (output) and experimental results (target) on three-dimensional graphics is specified in Figure 8.

**Figure 9:**
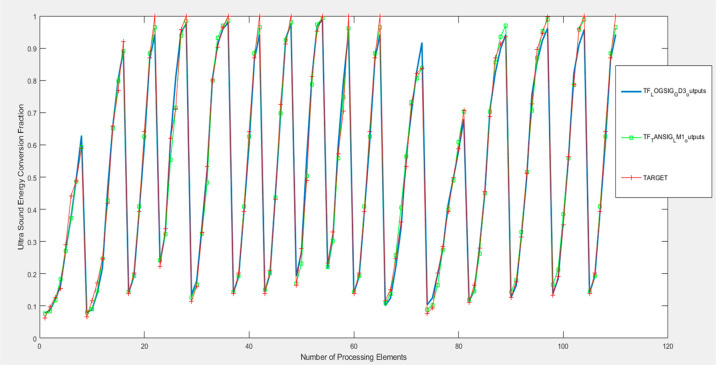
Comparison of the best ANN model results with experimental results.

A graphical comparison of ANN model results and experimental results by considering the variation of the values obtained according to the reaction time in the experimental study is given in Figure 9. From this graph, it has been determined that the ANN model results are very close to the experimental results.

## 4 Conclusions

This study consists of two stages. The first stage is the experimental stage, and the second stage is the modeling stage with artificial neural networks. The effect of ultrasound energy on the dissolution of the magnesite ore was investigated. In the second stage of the study, an artificial neural network model was created in accordance with this data set (Appendix 1). The input variables of the ANN model are time, particle size, reaction temperature, solid/liquid ratio, and amplitude ratio values. The output variable of the ANN model is the ultrasound energy fraction. In the ANN model, 110 data sets are divided into 70% training, 15% validation, and 15% test data sets. In this study, six different ANN models were created using two different learning algorithms and three different transfer functions.

In this study, Levenberg–Marquardt and Gradient Descent learning algorithms were used. As transfer functions, TANSIG, PURELIN, and LOGSIG, transfer functions in the neural network toolbox in the matlab software program, were used. The results of these models were compared with the experimental results (Appendix 1). It has been determined that the model established with the Levenberg–Marquart learning algorithm and the TANSIG transfer function gives the best result of the ANN model compared to the other models. The ANN model established with the Gradient Descent learning algorithm and the LOGSIG transfer function was determined to be the second model that gave the best results. The regression *R* value for the models’ performance indicator training data was determined as 0.99 after validation, and the regression *R* value for the test data was determined as 0.99. Accordingly, it has been determined that the ANN models give very close results to the real experimental results in estimating the ultrasound energy conversion fraction.

## Appendix

**Table A1:** Study Data Set, ANN Model Results

particle size (mesh)	time (da)	reaction temperature (°c )	solid/liquid ratio	amplitute speed	experimental ultra sound energy conversion fraction	LM 1 ANN predict	LM 2 ANN predict	LM 3 ANN predict	GD 1 ANN predict	GD 2 ANN predict	GD 3 ANN predict
10	3	20	0.008	50	0.063665	0.077	0.071	0.073	0.073	0.075	0.078
10	5	20	0.008	50	0.096684	0.084	0.073	0.078	0.078	0.078	0.086
10	10	20	0.008	50	0.12733	0.118	0.079	0.106	0.106	0.086	0.12
10	15	20	0.008	50	0.153857	0.183	0.088	0.155	0.155	0.1	0.164
10	30	20	0.008	50	0.289564	0.271	0.16	0.299	0.299	0.203	0.275
10	45	20	0.008	50	0.441885	0.373	0.375	0.394	0.394	0.465	0.374
10	60	20	0.008	50	0.488098	0.487	0.703	0.488	0.488	0.778	0.493
10	75	20	0.008	50	0.588901	0.593	0.909	0.595	0.595	0.937	0.629
15	3	20	0.008	50	0.066318	0.08	0.103	0.081	0.081	0.112	0.079
15	5	20	0.008	50	0.115602	0.091	0.112	0.093	0.093	0.122	0.09
15	10	20	0.008	50	0.171309	0.147	0.139	0.152	0.152	0.155	0.139
15	15	20	0.008	50	0.246702	0.247	0.181	0.247	0.247	0.204	0.218
15	30	20	0.008	50	0.419127	0.428	0.423	0.497	0.497	0.468	0.464
15	45	20	0.008	50	0.657871	0.651	0.747	0.635	0.635	0.78	0.659
15	60	20	0.008	50	0.769284	0.797	0.926	0.772	0.772	0.938	0.803
15	75	20	0.008	50	0.920489	0.89	0.982	0.9	0.9	0.985	0.893
20	3	20	0.008	50	0.137941	0.143	0.245	0.145	0.145	0.244	0.141
20	5	20	0.008	50	0.201606	0.194	0.276	0.198	0.198	0.274	0.191
20	10	20	0.008	50	0.3926	0.409	0.366	0.402	0.402	0.364	0.392
20	15	20	0.008	50	0.641955	0.625	0.473	0.612	0.612	0.47	0.602
20	30	20	0.008	50	0.870087	0.884	0.786	0.891	0.891	0.782	0.864
20	45	20	0.008	50	0.999825	0.965	0.94	0.97	0.97	0.938	0.942
25	3	20	0.008	50	0.222827	0.241	0.594	0.263	0.263	0.541	0.234
25	5	20	0.008	50	0.339546	0.322	0.638	0.349	0.349	0.587	0.326
25	10	20	0.008	50	0.620035	0.554	0.739	0.581	0.581	0.694	0.615
25	15	20	0.008	50	0.712461	0.715	0.82	0.752	0.752	0.784	0.81
25	30	20	0.008	50	0.958045	0.939	0.951	0.949	0.949	0.939	0.949
25	45	20	0.008	50	0.999825	0.983	0.988	0.985	0.985	0.985	0.976
20	3	14	0.008	50	0.115183	0.127	0.223	0.139	0.139	0.221	0.134
20	5	14	0.008	50	0.161396	0.166	0.25	0.185	0.185	0.248	0.178
20	10	14	0.008	50	0.328517	0.324	0.334	0.357	0.357	0.331	0.346
20	15	14	0.008	50	0.532775	0.483	0.437	0.542	0.542	0.432	0.526
20	30	14	0.008	50	0.801396	0.799	0.758	0.841	0.841	0.753	0.804
20	45	14	0.008	50	0.902565	0.932	0.93	0.948	0.948	0.928	0.912
20	60	14	0.008	50	0.964921	0.97	0.983	0.987	0.987	0.982	0.96
20	75	14	0.008	50	0.999825	0.985	0.996	0.996	0.996	0.996	0.979
20	3	20	0.008	50	0.137941	0.143	0.245	0.145	0.145	0.244	0.141
20	5	20	0.008	50	0.201606	0.194	0.276	0.198	0.198	0.274	0.191
20	10	20	0.008	50	0.3926	0.409	0.366	0.402	0.402	0.364	0.392
20	15	20	0.008	50	0.641955	0.625	0.473	0.612	0.612	0.47	0.602
20	30	20	0.008	50	0.870087	0.884	0.786	0.891	0.891	0.782	0.864
20	45	20	0.008	50	0.999825	0.965	0.94	0.97	0.97	0.938	0.942
20	3	30	0.008	50	0.137941	0.15	0.288	0.148	0.148	0.289	0.146
20	5	30	0.008	50	0.211099	0.202	0.323	0.206	0.206	0.323	0.2
20	10	30	0.008	50	0.430576	0.436	0.423	0.446	0.446	0.424	0.439
20	15	30	0.008	50	0.726283	0.697	0.536	0.694	0.694	0.536	0.69
20	30	30	0.008	50	0.91267	0.926	0.827	0.957	0.957	0.826	0.93
20	45	30	0.008	50	0.999825	0.981	0.953	0.991	0.991	0.953	0.975
20	3	40	0.008	50	0.164468	0.169	0.336	0.153	0.153	0.34	0.193
20	5	40	0.008	50	0.277417	0.231	0.376	0.22	0.22	0.379	0.267
20	10	40	0.008	50	0.488935	0.503	0.484	0.516	0.516	0.487	0.562
20	15	40	0.008	50	0.81089	0.788	0.598	0.788	0.788	0.601	0.81
20	30	40	0.008	50	0.952182	0.973	0.861	0.972	0.972	0.862	0.969
20	45	40	0.008	50	0.999825	0.994	0.964	0.989	0.989	0.964	0.988
20	3	20	0.004	50	0.233438	0.222	0.34	0.221	0.221	0.336	0.218
20	5	20	0.004	50	0.328935	0.303	0.38	0.304	0.304	0.375	0.308
20	10	20	0.004	50	0.572984	0.559	0.488	0.548	0.548	0.483	0.596
20	15	20	0.004	50	0.70562	0.748	0.602	0.731	0.731	0.596	0.795
20	30	20	0.004	50	0.999825	0.962	0.864	0.929	0.929	0.86	0.951
20	3	20	0.008	50	0.137941	0.143	0.245	0.145	0.145	0.244	0.141
20	5	20	0.008	50	0.201606	0.194	0.276	0.198	0.198	0.274	0.191
20	10	20	0.008	50	0.3926	0.409	0.366	0.402	0.402	0.364	0.392
20	15	20	0.008	50	0.641955	0.625	0.473	0.612	0.612	0.47	0.602
20	30	20	0.008	50	0.870087	0.884	0.786	0.891	0.891	0.782	0.864
20	45	20	0.008	50	0.999825	0.965	0.94	0.97	0.97	0.938	0.942
20	3	20	0.016	50	0.116719	0.109	0.133	0.11	0.11	0.134	0.101
20	5	20	0.016	50	0.151204	0.136	0.146	0.137	0.137	0.148	0.124
20	10	20	0.016	50	0.246702	0.257	0.191	0.244	0.244	0.193	0.22
20	15	20	0.016	50	0.360209	0.406	0.255	0.369	0.369	0.257	0.339
20	30	20	0.016	50	0.533194	0.563	0.557	0.563	0.563	0.558	0.566
20	45	20	0.016	50	0.724188	0.732	0.839	0.678	0.678	0.839	0.701
20	60	20	0.016	50	0.822339	0.806	0.957	0.801	0.801	0.957	0.826
20	75	20	0.016	50	0.837696	0.838	0.99	0.842	0.842	0.989	0.917
20	3	20	0.032	50	0.07733	0.089	0.072	0.094	0.094	0.072	0.104
20	5	20	0.032	50	0.093543	0.102	0.073	0.11	0.11	0.074	0.125
20	10	20	0.032	50	0.201606	0.164	0.079	0.183	0.183	0.081	0.198
20	15	20	0.032	50	0.285689	0.275	0.089	0.281	0.281	0.091	0.276
20	30	20	0.032	50	0.3926	0.401	0.165	0.435	0.435	0.17	0.414
20	45	20	0.032	50	0.498063	0.489	0.387	0.491	0.491	0.397	0.496
20	60	20	0.032	50	0.587086	0.608	0.715	0.624	0.624	0.723	0.582
20	75	20	0.032	50	0.706736	0.704	0.914	0.704	0.704	0.917	0.68
20	3	20	0.008	20	0.111414	0.119	0.165	0.134	0.134	0.176	0.116
20	5	20	0.008	20	0.164468	0.145	0.184	0.171	0.171	0.197	0.147
20	10	20	0.008	20	0.279651	0.263	0.245	0.304	0.304	0.263	0.282
20	15	20	0.008	20	0.455428	0.451	0.328	0.449	0.449	0.35	0.449
20	30	20	0.008	20	0.688168	0.703	0.653	0.694	0.694	0.676	0.711
20	45	20	0.008	20	0.870785	0.856	0.888	0.862	0.862	0.898	0.823
20	60	20	0.008	20	0.912531	0.935	0.971	0.952	0.952	0.974	0.897
20	75	20	0.008	20	0.935288	0.97	0.993	0.977	0.977	0.994	0.942
20	3	20	0.008	30	0.125654	0.144	0.188	0.13	0.13	0.196	0.126
20	5	20	0.008	30	0.175079	0.18	0.21	0.167	0.167	0.22	0.163
20	10	20	0.008	30	0.314415	0.329	0.281	0.305	0.305	0.294	0.319
20	15	20	0.008	30	0.511553	0.515	0.373	0.457	0.457	0.388	0.498
20	30	20	0.008	30	0.726841	0.707	0.702	0.727	0.727	0.714	0.756
20	45	20	0.008	30	0.896614	0.87	0.909	0.897	0.897	0.913	0.858
20	60	20	0.008	30	0.946597	0.953	0.977	0.972	0.972	0.978	0.924
20	75	20	0.008	30	0.996719	0.989	0.994	0.989	0.989	0.995	0.962
20	3	20	0.008	40	0.132635	0.166	0.214	0.136	0.136	0.219	0.138
20	5	20	0.008	40	0.19267	0.213	0.241	0.182	0.182	0.246	0.184
20	10	20	0.008	40	0.351553	0.385	0.321	0.36	0.36	0.327	0.367
20	15	20	0.008	40	0.563072	0.558	0.422	0.552	0.552	0.428	0.562
20	30	20	0.008	40	0.787435	0.786	0.746	0.846	0.846	0.75	0.821
20	45	20	0.008	40	0.960279	0.958	0.926	0.956	0.956	0.927	0.912
20	60	20	0.008	40	0.999825	0.989	0.982	0.989	0.989	0.982	0.958
20	3	20	0.008	50	0.137941	0.143	0.245	0.145	0.145	0.244	0.141
20	5	20	0.008	50	0.201606	0.194	0.276	0.198	0.198	0.274	0.191
20	10	20	0.008	50	0.3926	0.409	0.366	0.402	0.402	0.364	0.392
20	15	20	0.008	50	0.641955	0.625	0.473	0.612	0.612	0.47	0.602
20	30	20	0.008	50	0.870087	0.884	0.786	0.891	0.891	0.782	0.864
20	45	20	0.008	50	0.999825	0.965	0.94	0.97	0.97	0.938	0.942
